# Complex Segregation Analysis of Pedigrees from the Gilda Radner Familial Ovarian Cancer Registry Reveals Evidence for Mendelian Dominant Inheritance

**DOI:** 10.1371/journal.pone.0005939

**Published:** 2009-06-17

**Authors:** Bamidele O. Tayo, Richard A. DiCioccio, Yulan Liang, Maurizio Trevisan, Richard S. Cooper, Shashikant Lele, Lara Sucheston, Steven M. Piver, Kunle Odunsi

**Affiliations:** 1 Department of Preventive Medicine and Epidemiology, Loyola University Medical Center, Chicago, Illinois, United States of America; 2 Department of Cancer Genetics, Roswell Park Cancer Institute, Buffalo, New York, United States of America; 3 Department of Biostatistics, State University of New York at Buffalo, Buffalo, New York, United States of America; 4 School of Public Health and Health Professions, State University of New York at Buffalo, Buffalo, New York, United States of America; 5 Sisters of Charity Hospital, Buffalo, New York, United States of America; 6 Department of Gynecologic Oncology, Roswell Park Cancer Institute, Buffalo, New York, United States of America; 7 Department of Immunology, Roswell Park Cancer Institute, Buffalo, New York, United States of America; Ohio State University Medical Center, United States of America

## Abstract

**Background:**

Familial component is estimated to account for about 10% of ovarian cancer. However, the mode of inheritance of ovarian cancer remains poorly understood. The goal of this study was to investigate the inheritance model that best fits the observed transmission pattern of ovarian cancer among 7669 members of 1919 pedigrees ascertained through probands from the Gilda Radner Familial Ovarian Cancer Registry at Roswell Park Cancer Institute, Buffalo, New York.

**Methodology/Principal Findings:**

Using the Statistical Analysis for Genetic Epidemiology program, we carried out complex segregation analyses of ovarian cancer affection status by fitting different genetic hypothesis-based regressive multivariate logistic models. We evaluated the likelihood of sporadic, major gene, environmental, general, and six types of Mendelian models. Under each hypothesized model, we also estimated the susceptibility allele frequency, transmission probabilities for the susceptibility allele, baseline susceptibility and estimates of familial association. Comparisons between models were carried out using either maximum likelihood ratio test in the case of hierarchical models, or Akaike information criterion for non-nested models. When assessed against sporadic model without familial association, the model with both parent-offspring and sib-sib residual association could not be rejected. Likewise, the Mendelian dominant model that included familial residual association provided the best-fitting for the inheritance of ovarian cancer. The estimated disease allele frequency in the dominant model was 0.21.

**Conclusions/Significance:**

This report provides support for a genetic role in susceptibility to ovarian cancer with a major autosomal dominant component. This model does not preclude the possibility of polygenic inheritance of combined effects of multiple low penetrance susceptibility alleles segregating dominantly.

## Introduction

Established genetic risk factors for epithelial ovarian cancer (EOC) include the presence of an inherited mutation in one of the four ovarian cancer susceptibility genes, BRCA1, BRCA2, MSH2 or MLH1 [1]–[6]. However, not all families with a history of ovarian cancer will be carriers of any of these genes and, in those mutation-positive families, on average, only one-half of at-risk women will be carriers. In an effort to understand the role of genetic factors in the etiology of ovarian cancer, several studies have been carried out in different human populations [1], [5], [7]–[18]. These studies ranged from genetic epidemiological and segregation analyses [8], [14] that investigate mutations in specific genes by molecular genetics [10], [11], [13], [17], to risk and survival analyses [5], [12], [18] among families and pedigrees with affected relatives. Despite these studies, the mode of inheritance of susceptibility to ovarian cancer is not completely understood. In a recent study, members of 283 epithelial ovarian cancer families from the United Kingdom (UK) and the United States (US) were screened for coding sequence changes and large genomic alterations (rearrangements and deletions) in the BRCA1 and BRCA2 genes [19]. Of the deleterious mutations identified in the families, 37% and 9% were found in BRCA1 and BRCA2 genes respectively. Moreover, screening for MSH2 and MLH1 mutations in 77 cases of familial ovarian cancer, who previously tested negative for BRCA1 and BRCA2 mutations, revealed 2 cases with MSH2 mutations and none with a MLH1 mutation [20]. While these results indicate that BRCA1, BRCA2 and MSH2 are important susceptibility genes for ovarian cancer, it is also clear that other susceptibility gene(s) may exist.

Segregation analysis is often a starting point for family-based genetic studies of complex human diseases [21]. It helps to assess the possible genetic mode of segregation of disease by consideration of relevant hypothesis-based mathematical models. One advantage of segregation analysis is that it does not strictly require availability of genetic markers on study participants. Findings from segregation analyses are often used to formulate tailored research hypotheses on the disease under investigation, and/or to decide on the type of investigative effort on the disease. This study was therefore carried out to assess types of familial dependence in ovarian cancer, to investigate possible evidence of transmission of major gene(s) for ovarian cancer; and to determine the best mode of transmission for such major gene(s) in our data on 1919 pedigrees from the Gilda Radner Familial Ovarian Cancer Registry (GRFOCR). This study was intended to provide data on the nature of the genetic role in the apparent familial pattern of ovarian cancer susceptibility in this study population.

## Results

### Characteristics of GRFOCR members

**Figure 1:**
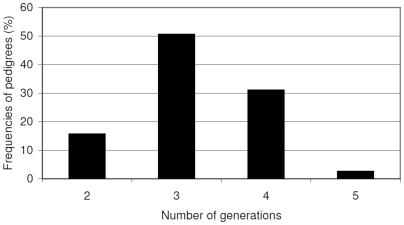
Plot showing distributions of number of generations in the 1919 pedigrees.

**Figure 2:**
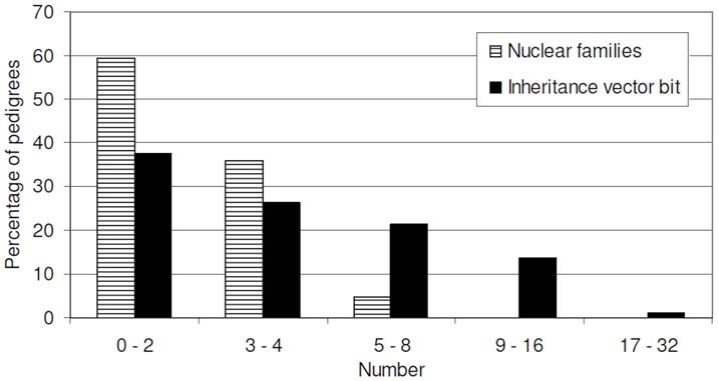
Distributions of numbers of nuclear families and inheritance vector bit in the 1919 pedigrees.

Table 1 shows the distribution of relationship types and total number of study subjects included in this study. Of the 7669 total number of individuals in the data, 6213 were females and 1456 males. Of the 6213 females, 3802 were affected and 2253 unaffected while affection status for 158 females was unknown. Individuals with unknown affection status were retained in the data to establish relationship within pedigrees, but were not used in the analysis because their phenotype values were set to missing. Overall, the data is composed of 15336 parent-offspring pairs, 4825 different sib-pairs broken down to sister-sister (n = 2900), sister-brother n = 1543), brother-brother (n = 382), and half sib (n = 8) pairs (Table 1). In the second- and third-degree relative categories, there were 9742 grandparental, 2709 avuncular and 4 cousin pairs. The number of generations per pedigree varies from 2 to 5 generations (Figure 1) which seem to account for the large range of number of nuclear families per pedigree and the distribution of inheritance vector bits seen in the data (Figure 2). Among the relationship pairs, there were 1062 parent/offspring, 935 sister/sister, 2 half sib, 161 grandparental and 272 avuncular concordant for ovarian cancer (Table 2).

**Table 1:** Distribution of relationship types in the study sample.

Relationship	Count
Proband	1919
Parent: Offspring	15336
Sib-pairs	4825
Sister: Sister	2900
Sister: Brother	1543
Brother: Brother	382
Halfsib	8
Grandparental	9742
Avuncular	2709
Cousin	4
Individuals	7669 (6647) ‡
Male	1456 (4025)
Female	6213 (2622)
Affected	3802
Unaffected	2253
Unknown	158

‡ Values in parentheses indicate number of dummy individuals used for the purpose of pedigree connections and who were not considered in analysis. These dummies were mostly pedigree founders.

**Table 2:** Distribution of ovarian cancer affection status among relationship pairs.

Pairs	Concordant affected	Discordant
Parent/Offspring	1062	5311
Sib-pairs		
Sister/Sister	935	1156
Half Sib	2	4
Grandparental	161	2943
Avuncular	272	1455
Cousin	0	3

The BRCA1, BRCA2, MSH2, and MLH1 mutation status of a subset of GRFOCR members was recently reported [19], [22]. In 137 GRFOCR families, the frequency of BRCA1 and BRCA2 mutations was 39%. In 77 of these families negative for a BRCA1 or BRCA2 mutation, the frequency of MSH2 or MLH1 mutation was 2.6% and 0%, respectively. These results confirm that BRCA1, BRCA2, MSH2 and MLH1 mutations do not fully account for familial predisposition to ovarian cancer.

### Segregation analyses indicate evidence for the familial transmission of a major gene in EOC

The parameter estimates and test statistics from the complex segregation analyses are presented in Table 3. All analyses were restricted to females by setting the penetrance (the probability that an individual will be affected) for males equal to zero. To account for presence of other cancer types of interest, we incorporated in our models additional covariate, but because there were only a few individuals with such other types of cancers, the inclusion or exclusion of the covariate had no effect on the models (data not shown). This covariate was subsequently dropped from all analyses reported in this study.

**Table 3:** Parameter estimates from segregation analysis of ovarian cancer in 1919 proband-ascertained pedigrees.

			Model parameters												
Hypothesis	Model No.		Transmission probabilities			Susceptibilities			Residual Associations§						
		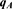	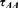	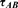	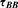	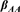	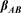	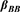	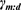	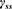	df§	-2 ln L	AIC	χ^2^ (df)	P
Sporadic	1	[0] †	–	–	–	−382.91	[ = β_AA_]	[ = β_AA_]	–	–	1	5731.20	5733.20	115.27 (7)	<.001
Sporadic with FA	2	[0]	–	–	–	−382.97	[ = β_AA_]	[ = β_AA_]	0.04	0.68	3	5661.95	5667.95	46.02 (5)	<.001
Major Gene only	3	0.50	M^‡^	M	M	−326.08	−407.14	−326.08	–	–	4	5749.69	5757.69	133.76 (4)	<.001
Codominant	4	1.00^*^	M	M	M	−382.97	3.60	5.76	0.04	0.68	5	5661.95	5671.95	46.02 (3)	<.001
Dominant	5	0.21	M	M	M	−389.85	[ = β_AA_]	−382.37	0.07	1.61	5	5617.23	5627.23	1.3 (3)	0.729
Recessive	6	0.94	M	M	M	−383.31	−255.96	[ = β_AB_]	−0.24	0.62	5	5650.12	5660.12	34.19 (3)	<.001
Additive	7	1.00^*^	M	M	M	−382.97	−382.96	10.56	0.04	0.68	4	5661.95	5669.95	46.02 (4)	<.001
Decreasing	8	0.00^*^	M	M	M	7.91	7.51	−382.97	0.04	0.68	5	5661.95	5671.95	46.02 (3)	<.001
Increasing	9	1.00^*^	M	M	M	−382.97	1.98	5.76	0.04	0.68	5	5661.95	5671.95	46.02 (3)	<.001
Environmental	10	0.72	[0.72]	[0.72]	[0.72]	−392.71	−307.48	−256.07	−1.00	1.00	6	5731.20	5743.20	115.27 (2)	<.001
Tau AB free	11	0.74	[1]	0.70	[0]	−429.79	5.00	8.00	0.99	0.99	7	5746.38	5760.38	130.45 (1)	<.001
General	12	0.49	0.90	0.57	1.00^*^	−393.66	0.23	0.38	1.00	1.00	8	5615.93	5631.93	–	–

† Parameters in square brackets were fixed at the values indicated; **^‡^**M indicates Mendelian transmission: τ_AA_ = 1.0, τ_AB_ = 0.5, τ_BB_ = 0.0.

§ The meaning of γ parameters is as follows: ^γ^_md_ represents mother/daughter residual association; ^γ^_ss_ represents sister/sister residual association; ^*^Parameter hit bound; **^§^**No. of independent parameters: (no. of parameters in model) – (no. of parameters fixed at boundary) – (no. of dependent and or fixed parameters); Chi-square is defined as (-2 ln L) of the data under the specific hypothesis minus (−2 ln L) of the data under the general model.

To determine support for familial or residual association in the data, firstly we compared three sporadic models, each having different type of familial association – parent-offspring, sibling or both parent-offspring and sibling. The model with both parent-offspring and sibling residual association fitted the data better than either of the other two (results not shown). Secondly, we then compared a sporadic model without residual association parameter (model 1) with the sporadic model that included both offspring and sibling residual association (model 2). The model with familial association significantly fitted the data better than the one without (Model 2 vs. 1, 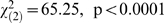), thereby providing support for the existence of familial association in the data and justification for estimation of familial association parameters in the subsequent models.

Next, we tested the hypothesis of no major gene by comparing the sporadic model 2 (sporadic with FA) with the general or full model (model 12). The sporadic model was strongly rejected (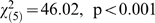), thus providing support for the existence of a major gene. The hypothesis of a major gene only was tested by comparing model 3 and model 12. Again, the hypothesis of a major gene only was rejected (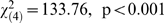). To investigate possible transmission of the major gene, the hypothesis of “no type-specific transmission” was assessed by comparing the environmental model (model 10) in which transmission parameters are constrained equal to allele frequency, with the general model (model 12) in which transmission and allele frequency parameters were estimated. This hypothesis of no type-specific transmission was also rejected (Model 10 vs. 12: 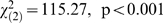). The rejection of the environmental model is an indication of transmission of major gene type-specific.

To establish the evidence for segregation of major gene (s), the hypothesis of Mendelian transmission must fail to be rejected in addition to rejection of both hypotheses of “no major effect” and “no transmission of major effect” [23], [24]. Since the last two criteria have been met, the hypothesis of “Mendelian transmission” was therefore tested by comparing all the different types of Mendelian models (Models 4–9) with the general model. The dominant Mendelian model (model 5) could not be rejected (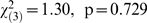), thereby providing supporting evidence for the transmission of major gene with a susceptibility allele frequency of 0.21.

### EOC segregates in a Mendelian dominant fashion

To further determine if the transmission probabilities of the major gene in the data conform with Mendelian mode, we compared the model in which only the 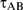 was estimated (model 11) with the Mendelian dominant model. The Akaike Information Criterion (AIC) values indicate that the dominant model is a better-fitting model (Model 5 (AIC = 5627.23) vs. Model 11 (AIC = 5760.38)). Because BRCA1, BRCA2, MSH2, and MLH1 mutations are known to be associated with elevated risk of ovarian cancer and are transmitted in an autosomal dominant fashion, we investigated if the observed evidence for dominant inheritance was driven by a single or multiple loci. Using the dominant Mendelian model, we fitted several polygenic mixed models with a parameter for varying number of loci. The AIC value for the dominant Mendelian model assuming three polygenic loci was the smallest (6413.02) compared to the other models assuming two (AIC = 6441.18) or four (AIC = 6424.61) polygenic loci. It is therefore not unlikely that the observed mode consists of polygenic inheritance of combined effects of multiple low penetrance susceptibility alleles segregating dominantly. The results of this study thus provide evidence for genetic role in the etiology of ovarian cancer by showing support for Mendelian dominant mode of segregation of susceptibility to epithelial ovarian cancer.

## Discussion

We present here results from complex segregation analysis of ovarian cancer susceptibility. We analyzed 1919 pedigrees derived from the Gilda Radner Familial Ovarian Cancer Registry (formerly referred to as the Familial Ovarian Cancer Registry) at the Roswell Park Cancer Institute (RPCI), Buffalo, New York, USA. Each pedigree was ascertained through affected proband and because of the inclusion criteria discussed in details under the materials and methods above, our sample was a little more enriched with affected individuals possibly more than would have been seen in unselected samples. In this study, we restricted analysis to female pedigree members since ovarian cancer does not occur in men. To achieve this restrictive analysis, we treated all males as unaffected and then set the penetrance for susceptibility to ovarian cancer as zero for males. To assess the hypothesis of no familial association, we compared the likelihood of the sporadic model without familial components with that in which parameters for both parent-offspring and sib-sib were estimated. Since the analyses were restricted to only females, the parent-offspring and sib-sib parameters are interpreted as mother-daughter and sister-sister, respectively. The model with familial association parameters provided better fit than it's counterpart that did not include familial association components. The better fitting of the model thus provided evidence of familial association in susceptibility to ovarian cancer.

We also investigated the mode of inheritance of ovarian cancer susceptibility; whether it was sporadic, environmental, or Mendelian. Although the observed aggregation of affection status in the data points to familial association and is consistent with an inheritance basis, we had to evaluate all relevant possible genetic models to ascertain the most likely mode of inheritance. We estimated the disease allele frequency alongside the susceptibility and transmission parameters depending on specific assumption for the models. Because all the hypothesis-based models were hierarchical in setting, since they were nested in the most general model, we based our statistical inferences on likelihood ratio test. Our data indicate that (i) the sporadic model of segregation of a major gene with familial association must be rejected when compared to the general with familial component (ii) the hypothesis of Mendelian transmission must be accepted in favor of general transmission, and (iii) the hypothesis of no transmission of a major gene must be rejected when compared to the general transmission. We showed that both the sporadic and environmental (no transmission model) were rejected with p-values much less than 0.001, while the autosomal dominant Mendelian model could not be rejected against the most general model (Table 3).

In a previous study, segregation analysis of 112 high risk ovarian cancer families found that BRCA1/2 mutations accounted for only about one half of familial ovarian cancer (5). However, there was little evidence that other major high-penetrance ovarian cancer susceptibility genes explain the residual familial ovarian cancer [5]. Although non-BRCA1/2 risk for ovarian and breast cancers may be transmitted by different modes, a segregation analysis of 858 families of early onset breast cancer reported a residual dominantly inherited risk of breast cancer besides the risk derived from mutations in BRCA1/2. [25]. In the GRFOCR, BRCA1/2 mutations were found in 39% of 137 families tested and MSH2 mutations were found in 2.6% of 77 families tested [19]. The limited number of families tested precluded investigating the possible influence of BRCA1/2 mutations on the observed dominant Mendelian mode of segregation of ovarian cancer in the current study of 1919 GRFOCR families. Despite this limitation, the investigations using the GRFOCR provide evidence supporting a dominant mode of segregation of susceptibility to ovarian cancer and the possibility of ovarian cancer susceptibility genes besides BRCA1, BRCA2 and MSH2.

## Materials and Methods

### Ethics Statement

The Gilda Radner Familial Ovarian Cancer Registry: Family and Medical History and Biosample Resource (CIC 95–27) protocol has been reviewed and approved the Roswell Park Cancer Institute IRB Board.

#### Study subjects and characteristics of family members

Study subjects for this study were derived from the Gilda Radner Familial Ovarian Cancer Registry (formerly referred to as the Familial Ovarian Cancer Registry). The Gilda Radner Familial Ovarian Cancer Registry (GRFOCR) is a self-referred Registry of families with two or more ovarian cancer cases in blood relatives. It was established in 1981 at the Roswell Park Cancer Institute by Dr. M. Steven Piver to study the incidence of familial ovarian cancer [26]–[29]. The primary function of the Registry is to receive family cancer information voluntarily contributed throughout the United States by ovarian cancer patients, referring and concerned physicians, concerned women, and patients of the Roswell Park Cancer Institute (RPCI) Gynecologic Oncology Department. The objectives of the Registry include (i) obtaining detailed family histories from individuals who are apparently from families with two or more cases of ovarian cancer or a syndrome possibly related to ovarian cancer; (ii) documenting through medical records and through pathologist review of tissues the occurrence of cancer; (iii) collecting, processing and storing biological samples, when possible, from Registry participants; and (iv) making the information and biological samples available for research under Institutional Review Board approved research protocols.

#### Recruitment of subjects

Registry participants are recruited through probands or index persons meeting at least one of the following criteria: (i) family history of two or more cases of ovarian cancer; (ii) family history of one case of ovarian cancer and two cases of cancer at any other site; (iii) family history of at least one female with two or more primary tumors with one of the primaries being ovarian cancer; (iv) family history of two or more cases of cancer with at least one case being ovarian cancer, and the other cancer considered to be of early onset (≤45 years old). In addition to meeting at least one of the preceding criteria, each participant is required to sign a consent form. Individuals unable to give consent as a result of mental, intellectual, or cognitive deficits are excluded, however, such could appear in the Registry through collection of family history data but without collection of bio-samples from them. Subjects for this study consisted of 7669 adult members from 1919 different pedigrees from the GRFOCR. The families included in the present study comprised of 1412 families ascertained to have two or more cases of ovarian cancer; 17 families with one case of ovarian cancer and two cases of cancer at other sites; and 490 families in the category of those having at least one female with more than one primary tumors with one of the primaries being ovarian cancer or having more than one case of cancer with at least one case being ovarian cancer and the other cancer considered to be of early onset.

#### Data collection

Data are collected through Family History Forms completed by subjects. In addition, subjects give authorization to release medical records and archival tissues (where available). Both the completed Family History Form and retrieved medical records are reviewed to ascertain eligibility before subjects are entered into the Registry. Information collected on individuals who do not meet inclusion criteria at the time of collection are not entered into the Registry, and are either destroyed, placed in an inactive locked file, or returned to the individuals upon request. Following formal entry into the Registry, the individuals are provided with an epidemiologic survey form for collection of detailed epidemiologic data and a blood donation form for biosample collection. Permission to invite relatives is also requested from Registry participants and letter of introduction sent to relatives for whom permission to invite is granted. Invited relatives who accept to participate are also asked to sign a consent form after which they are asked to complete voluntarily all necessary data and biosample collection forms.

#### Construction of pedigrees

Based on information collected through the Family History Form, we established family and pedigree relationship of every subject. Pedigrees used in this study were constructed from relatives ascertained through probands using computer codes written and implemented in SAS [30] as macros and the resulting established pedigree relationships were checked and corrected for possible errors using MADELINE [31] software package. Where necessary, dummy individuals were added to families for the purpose of connecting relatives within pedigrees, and the affection status for such dummy individuals was set to missing and thus they were not used in the analyses. A total of 7669 real pedigrees members and 6647 connecting dummy persons were included in this study.

#### Statistical analysis

In the present study, data used included information on (i) sex, (ii) ovarian cancer affection status defined as affected, unaffected or unknown; (iii) information on affection status for other cancer site such as breast, pancreas and uterus/endometrium, also defined as affected, unaffected or unknown; and (iv) family/pedigree relationships. Estimation of the distributions of relationship types and ovarian cancer affection status among relationship pairs were performed using the Statistical Analysis for Genetic Epidemiology (SAGE) program PEDINFO, version 5.2 [32]. Although analyses were constrained to female pedigree members, male relatives had to be included for the purpose of defining pedigree relationships.

To account for proband ascertainment, ascertainment correction was applied in all segregation analyses by conditioning each pedigree's likelihood on the affection status of the proband.

#### Segregation Analysis

To explore the mode of familial transmission of susceptibility to ovarian cancer, we performed complex segregation analysis using the maximum likelihood method to estimate the parameters in each of the hypothesis-based mathematical models examined. Since the presence of a BRCA1 or BRCA2 mutation status does not preclude the presence of additional susceptibility gene(s) which could contribute to disease penetrance, mutation positive patients were therefore not excluded from this analysis. The SEGREG program of SAGE, version 5.2 [32] under Linux operating system was used to fit each model. For each model, we assumed that the presence (or absence) of the putative disease allele influences susceptibility to ovarian cancer, and then applied the regressive multivariate logistic model for binary trait as described by Karunaratne and Elston [33]. This approach enabled us to include available covariates of interest in the fitted models. The fitted models assumed that, conditional on the phenotype and the major type of any individual who belongs to two nuclear families, the likelihoods for those two nuclear families are independent. Therefore, the marginal probability (or susceptibility) that any pedigree member has a particular phenotype is the same for all members who have the same values of any covariates in the model. This susceptibility given by the cumulative logistic function as
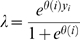
where *y_i_* is the affection status phenotype of the *i-th* individual; *θ_(i)_* is the logit of the susceptibility for the *i-th* individual which is defined as
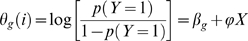
where *β* is the baseline parameter; *g* is the latent genetic “type” [34] or “ousiotype” [35]; and *X* is the covariate vector. Under a major locus model with two alleles, A and B, A being the susceptibility allele, the three types correspond to genotype g = AA, AB or BB transmitted according to Mendelian mode. The corresponding baseline parameters for susceptibility are then 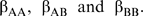 The transmission parameters represented as 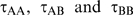 in each model are the conditional probabilities that a parent of a given genotype transmits the susceptibility allele A to the offspring [36], [37]. The transmission parameters and the allele frequency parameter, 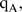 for the susceptibility allele are estimated alongside the three baseline parameters for susceptibility 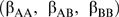 in each model depending on the specified assumption. For example, under the assumption of Mendelian inheritance, the transmission parameters are constrained to 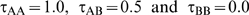. The following sporadic, environmental and genetic models were considered in assessing type of familial association and possible evidence of transmission of major effect.

1. Sporadic or no major gene model. In this model, both familial association (FA) (i.e. father-mother (FM), mother–offspring (MD), father–offspring (FO) or sibling (SS)), and transmission of major gene (MG) (i.e. 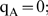 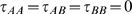) are not assumed. There is only one baseline parameter (i.e. 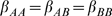) which is interpreted as the natural logarithm of the odds of susceptibility versus non-susceptibility to ovarian cancer in the absence of other factors.
2. Sporadic model with familial association. This model includes estimation of parameters for familial association (parent-offspring (PO) and sibling) in the absence of transmission of a major gene. Three different models – first with only parent-offspring parameter, second with only sibling parameter, and third with both parent-offspring and sibling parameters, were fitted.
3. Major gene without familial association model. This model assumes the transmission of a major gene but no familial association. Here the susceptibility allele frequency is estimated while the transmission parameters are constrained to the Mendelian mode.
4. Mendelian codominant model with familial association. This assumes transmission of a major gene and familial association. In this and all Mendelian models tested in this study, transmission parameters were constrained to the Mendelian mode as 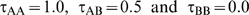. The allele frequency, 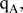 familial or residual associations, 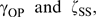 and baseline susceptibility, 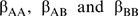 were estimated in this and the other Mendelian models tested.
5. Mendelian dominant model which is similar to codominant model above, except that baseline susceptibility parameters for genotypes AA and AB are constrained equal as 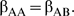

6. Mendelian recessive model in which baseline susceptibility parameters for genotypes AB and BB are constrained equal to each other as 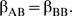

7. Mendelian additive model in which baseline susceptibility parameter for genotype AB is constrained to be intermediate of those of AA and BB as 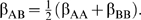.
8. Mendelian decreasing model includes the assumption of decreasing susceptibility with the maximum and minimum susceptibility parameters constrained to genotypes AA and BB, respectively, as 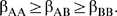.
9. Mendelian increasing model is the reverse of the above decreasing model with the baseline susceptibility parameters constrained as 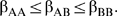

10. Environmental model in which no transmission of susceptibility allele is assumed. This is a non-Mendelian transmission model in which all the transmission probabilities are set equal to the allele frequency as 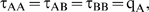 but all three susceptibility parameters 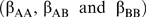 are estimated. This model assumes that the observed familial association and segregation are both due purely to non-transmissible environmental effects and not any major gene.
11. Tau AB free model in which transmission parameters for genotypes AA and BB are constrained to 1 and 0, respectively, while parameter for AB is estimated within 0–1 range as 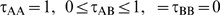.
12. General non-Mendelian model in which all parameters are estimated. As a result, all other models are nested in the general or full model and thus the general model is used as the baseline to compare all other models in this study.

In the above models 3 to 11 where major gene is assumed, we also assumed that the genotype frequencies are in Hardy-Weinberg proportions (i.e, 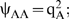 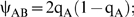 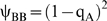). Also, because ascertainment was through probands, we corrected for ascertainment bias in each model by conditioning the likelihood of each pedigree on the affection status of the proband.

For testing the different hypotheses represented by the models, we used the likelihood ratio test (LRT). Since the models are hierarchical, we tested each submodel against the general model by using the test statistic computed as minus twice the difference between the natural log likelihood of the general model and that of the specific submodel. This statistic is asymptotically distributed as chi-square distribution with degree of freedom equal to the difference in the number of parameters estimated in both models. Using this test, a significant chi-square indicates that the submodel tested can be rejected at the given alpha level, which means the hypothesized model does not fit the data. For comparison of non-nested models such as sporadic models or Mendelian models, we used the Akaike Information Criterion (AIC) values [38] to select the most parsimonious model for the data. The AIC for any model is defined as [–2ln(L)+2(number of parameters estimated in the model)]. The model with the smallest AIC is judged the best-fitting model for the data.
